# Evaluation of morphometric traits and productive and reproductive performance of Sipli sheep in Punjab, Pakistan

**DOI:** 10.1371/journal.pone.0355695

**Published:** 2026-08-25

**Authors:** Aqsa Rubab, Mushtaq Hussain Lashari, Musarrat Abbas Khan, Mubarra Anam, Shazia Tahreem

**Affiliations:** 1 Department of Zoology, The Islamia University of Bahawalpur, Pakistan; 2 Department of Animal Breeding and Genetic, The Islamia University of Bahawalpur, Pakistan; Universidade Federal de Mato Grosso do Sul, BRAZIL

## Abstract

The study evaluated the morphometric traits, productive, and reproductive performance of Sipli sheep in Punjab, Pakistan, aiming to provide a baseline for breed characterization, management, and breeding strategies. The study was conducted at the Sheep Breeding Centre, involving the collection of morphometric data, productive traits (wool and milk), and reproductive parameters. Morphometric measurements, including body length, height at withers, and heart girth, were taken on 35 rams and 47 ewes. Wool samples (n = 33) were collected bi-annually from the same sheep during autumn and spring to assess fiber diameter, length, staple length, and wool yield. Milk yield and composition were analyzed from 14 ewes. Reproductive data, including pregnancy rates, birth type, and lambing rates, were collected during the spring and autumn seasons of 2021 and 2022. The data were analyzed using descriptive statistics, Pearson’s correlation, and ANOVA with Duncan’s multiple range test, in addition to evaluating four non-linear growth models (Brody, Von Bertalanffy, Gompertz, and Logistic) for growth curve analysis. Results revealed that rams had significantly larger body dimensions than ewes. The study showed a highly significant (p < 0.001) positive correlation between body length and diagonal body length (r = 0.814) and height at withers (r = 0.774) in rams. In lambs, body length increased with age, with a substantial reduction in the coefficient of variation, from 12.22% at 1–3 months to 8.81% at 10–12 months. The Brody model provided the best fit (R²adj = 0.6267; RMSE = 14.941) for growth curve analysis. Male lambs showed consistently higher weights than female lambs (p < 0.005) across all ages. Rams had a significantly higher medullated fiber content (19.27%) compared to ewes (12.56%), with autumn-sheared wool exhibiting thicker fiber diameters (34.94 µm). The pregnancy rate was 78% in the spring of 2021 and significantly reduced to 53.06% in the autumn of the same year. The highest lambing rate of 143.48% was observed in spring 2022. The study concluded that Sipli sheep demonstrate significant sexual dimorphism and the Brody model effectively described the growth dynamics in this breed. Environmental factors like the year of birth and seasons significantly impacted growth and reproduction. Male lambs and single births showed improved growth, with seasonal effects on wool production and significant variations in reproductive traits. These findings provide a basis for breeding practices by considering key phenotypic characters and their association with body weight. In the future, it is recommended that environmental effects should be included in selection programs to optimize growth performance, alongside further genetic studies.

## 1. Introduction

The livestock sector plays a vital role in Pakistan’s economy, contributing significantly to the national GDP and providing livelihoods for millions, especially in rural areas [1,2]. Sheep farming, in particular, offers essential resources like meat and wool, making it a cornerstone of agricultural sustainability. In the Punjab province, with its considerable sheep population [2], these animals are pivotal for household income and nutritional needs [3]. Globally, sheep are a major source of meat, ranking fourth in consumption, and also provide valuable wool and milk with superior nutritional properties and lesser allergenicity than cow’s milk [4]. However, the conservation of diverse sheep breeds is crucial, especially given the threat of genetic resource loss in the face of global challenges like climate change [5].

Understanding breed characteristics through morphometric analysis is fundamental for effective management and breeding programs. Such analyses can reveal variations across populations and identify key traits for improvement [6]. The increasing demand for animal protein necessitates research into growth traits, which can be enhanced through genetic selection and breeding strategies [7]. These strategies often rely on estimated breeding values (EBV) to identify animals with superior genetics, thereby increasing productivity [8].

Within Pakistan, indigenous breeds like Lohi and Kajli are well-adapted to local conditions [9]. The Sipli sheep, a breed native to the Cholistan desert region of Southern Punjab, is known for its unique characteristics and is a valuable resource for wool and mutton production [10,11]. Despite its importance, there is a lack of scientific literature specific the Sipli breed’s morphometric, productive, and reproductive traits. This lack of information limits the implementation of effective breeding and management strategies aimed at improving productivity and conserving this genetic resource. Therefore, this study aims to address this gap by comprehensively evaluating the Sipli sheep breed for morphometric traits and their correlation with body weight, productive and reproductive performances. The findings will serve as a foundation for future breeding strategies, conservation efforts, and management protocols, ultimately enhancing the productivity and profitability of Sipli sheep farming in Pakistan.

## 2. Materials and methods

### 2.1 Experimental site

The present study was conducted at the Sheep Breeding Centre, which is managed by the Department of Animal Breeding and Genetics within the Faculty of Veterinary and Animal Sciences at The Islamia University of Bahawalpur. This study was conducted with approval from the Board of Faculty and Advanced Studies & Research Board of The Islamia University of Bahawalpur (Approval No. 1112/AS&R, dated 13-11-2023). This location provided the necessary infrastructure and resources for the duration of the study.

### 2.2 Animal selection and grouping

The investigation focused exclusively on the Sipli sheep breed. These animals were maintained at the aforementioned Sheep Breeding Centre. The sheep population was systematically categorized into six groups, based on both age and sex. All animals were distinctly identified using individual tagging, ensuring precise data collection and tracking throughout the study period.

### 2.3 Housing and management

The Sipli sheep were housed in designated stalls, where they received a controlled diet. The diet consisted of chopped, fresh-cut seasonal forage, supplemented by a concentrate feed containing 15% crude protein. In addition, wheat straw and maize silage were provided to complement the diet. The management protocol ensured the availability of clean drinking water at all times. Consistent monitoring and tagging of each animal, provided data accuracy.

### 2.4 Morphometric measurements

This experiment was designed to determine the morphometric characteristics of Sipli sheep, drawing upon both quantitative and qualitative data. The sheep were categorized according to age and sex, as described previously. Data collection was performed following established guidelines provided by FAO (12) for phenotypic characterization.

#### 2.4.1 Quantitative data collection.

The quantitative data collection involved a series of measurements designed to characterize the morphometric traits of the Sipli sheep. These measurements were conducted monthly and employed a measuring tape and digital weighing scale, following established guidelines [12]. The study encompassed a range of traits, including body length (BL), defined as the distance from the base of the neck to the base of the tail; body weight (BW), representing the total weight of the sheep; heart girth (HG), the circumference of the chest measured just behind the front legs; and paunch girth (PG), the circumference of the abdominal area, taken at the level of the paunch. Further measurements included height at withers (HW), which is the distance from the ground to the highest point of the withers; height at sacrum (HS), which is the distance from the ground to the highest point of the sacrum; rump length (RL), the distance from the hip joint to the base of the tail; and rump width (RW), measured across the hip joints. The tail length (TL), head length (HL) (from the tip of the nose to the back of the head), and head circumference (HC) were also measured. Additional traits included ear length (EL), ear width (EW), neck length (NL), diagonal body length (DBL), pin width (PW), thurl length (ThL), teat length (TEL), teat diameter (TED), testes length (TsL), and testes diameter (TsD).

#### 2.4.2 Qualitative data collection.

In addition to quantitative measures, qualitative traits were recorded monthly. These included the color of the head, coat, and nasal bridge. Visual analysis was conducted to assess these traits, and frequency distributions were calculated to explore phenotypic diversity.

### 2.5 Environmental factors influencing growth

The aim of this experiment was to examine the influence of various environmental factors on the growth traits of Sipli sheep. The investigated factors included season of birth (categorized as spring, from March to September, and autumn, from October to February), sex (male and female lambs), birth type (single or twin births), and year of birth. Data collected included animal identification, birth weight, and body weights adjusted to 120, 180, 270, and 365 days of age, respectively. Adjustment of weights to specific ages was done using the following formula, adapted from Sharif, Ali [13]:

- z = [b + (w-b) /a] × d

Where:

- z = adjusted weight for specific days of age (120, 180, 270, and 365)
- b = birth weight
- w = weight recorded at 4, 6, 9, and 12 months
- a = age in days at the measurement of weight at 4, 6, 9, and 12 months
- d = 120, 180, 270, and 365 days accordingly for each trait.

### 2.6 Non-linear models to describe growth curves

This study aimed to identify the most suitable non-linear model to describe the growth curve of Sipli sheep. Body weights of Sipli lambs were recorded fortnightly, from birth up to 1 year of age. Only individuals with complete body weight data from birth to 12 months (comprising 25 observations per animal) were included in the final analysis. Any animal with missing data was excluded. Age and weight data were analyzed using four non-linear models: Gompertz, Von-Bertalanffy, Brody, and Logistic, all implemented using the easyreg package within R software. The mathematical representations of the models are as follows, following Sharif, Ali [14]:

- **Brody model:** y = A× [1 – B × exp (-k × T)]
- **Logistic model:** y = A× [1 + B × exp(-K × T)]-M
- **Gompertz model:** y = A × exp [-B × exp (-K × T)]
- **von Bertalanffy model:** y = A× (1 – B × exp(-K×T)^3^

Where:

- y = live weight at T (age in days)
- A = asymptotic weight
- B = rate of gain from birth to asymptotic weight
- K = rate of maturity
- M = trajectory shape determinant.

The performance of each model was evaluated using the Akaike’s Information Criterion (AIC), Bayesian Information Criterion (BIC), adjusted coefficient of determination (R²adj), and Root Mean Square Error (RMSE). The mathematical expressions for these criteria, following Bangar et al. (2018) and Lupi et al. (2015) are:

- R² = 1 – (RSS/TSS)
- R²adj = 1 – ((n-1)/(np))(1- R²)
- AIC = n ln (RSS/n) + 2p
- RMSE = √(RSS/(n-p-1))
- BIC = n ln (RSS/n) + pln(n)

Where:

- R² = pseudo R² determinative coefficient
- TSS = total sum of squares
- RSS = residual sum of squares
- p = parameters in the model
- n = number of observations
- ln = logarithm-function.

The optimal model for describing the growth curve of Sipli sheep was determined to be the one exhibiting the highest R²adj value and the lowest values for AIC, BIC, and RMSE.

### 2.7 Productive traits

#### 2.7.1 Wool quality and quantity traits.

**Fig 1:**
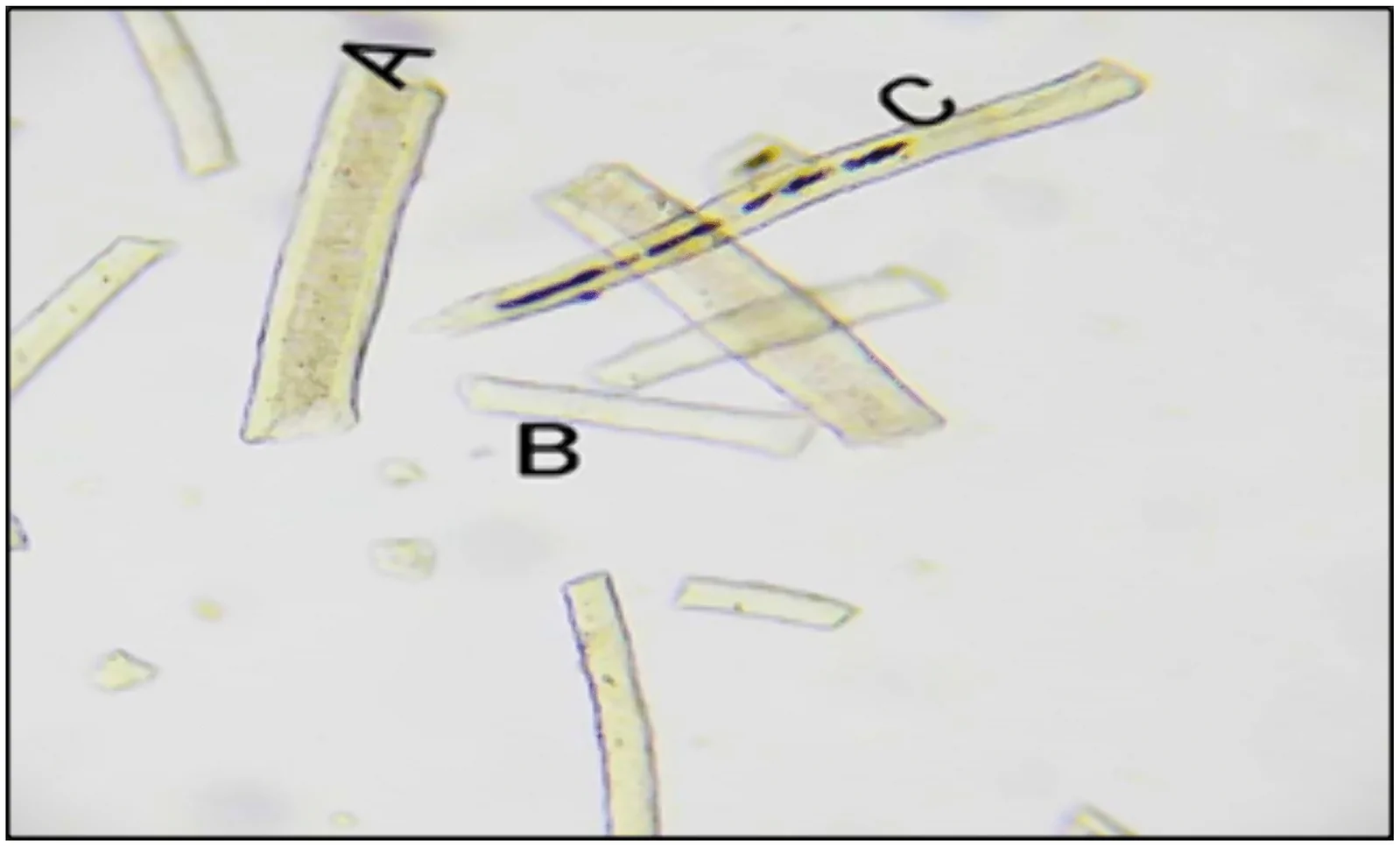
Types of fiber (A, modullated B, non-medullated (true fiber) and C, heterotypic).

This component of the study focused on assessing the wool quantity and quality traits of Sipli sheep in Punjab, Pakistan. Thirty-three sheep were sampled bi-annually during both the autumn and spring shearing seasons, resulting in 66 wool observations. Following collection, the samples were transported to the animal breeding and genetics laboratory, where they were stored in a dry, clean environment at room temperature. The analysis involved assessing traits, including staple length (cm), fiber length (cm), fiber diameter (µm), and other morphometric measurements. The study also considered various fiber types (Fig 1), including true fibers, heterotypic fibers, and the percentage of medullated fibers, alongside wool weight (kg), utilizing methodologies described by Singh, Gahlot [15].

Non-genetic factors such as gender (Ram and Ewe), season of birth (Spring, Winter, Autumn, Summer), season of shearing (Autumn, Spring), and animal age (1, 2, 3, and older than 3 years) were analyzed to understand their influence. Wool quality traits were assessed using the least squares methodology as described by Koonce [16], with the following model:

- Yijk = μ + Si + Pj+Ak +Gl+ eijkl

Where:

- Yijkl = Observation on the wool quality trait for the kth individual in the jth year, ith season of birth, kth age group and lth gender.
- μ = Overall mean
- Si = Fixed effect of ith season of birth (i = Spring, Winter (Oct–Feb), Autumn, Summer (Mar–Sep)).
- Pj = Fixed effect of jth season of shearing (j = Winter (Oct–Feb), Summer (Mar–Sep)).
- Ak = Fixed effect of the kth age group (k = 1, 2, 3, more than 3 years of age).
- Gl = Fixed effect of the lth gender (l = Male, Female).
- eijkl = error associated with each observation and assumed to be normally and independently distributed with mean zero and variance ó2 e (0, ó2e).

#### 2.7.2 Milk yield and composition.

This investigation examined the milk yield and composition of Sipli sheep (n = 14) in 2022. Milk samples were collected fortnightly, starting on the fifth day after lambing and continuing until the 90th day of lactation. The ewes were separated from their lambs the evening before sampling to ensure that their udders were full the next morning. Milk samples were collected through hand milking directly into sterile bottles, following the procedure outlined by Dzidic, Rovai [17]. Wool yield was measured utilizing a digital precision scale, ensuring consistent monitoring throughout the study. Subsequently, milk samples were immediately stored in ice and delivered to the Animal Nutrition Laboratory within the Department of Animal Nutrition. Milk compositional and physical properties were analyzed using a Lactoscan milk analyzer MCCW- V1 (MILKOTRONIC LTD, BULGARIA), which provided rapid and accurate measurements of milk components, thus increasing the reliability of the data. The measured parameters included salts, protein, fat, lactose, SNF percentage, sample temperature (°C), freezing point (°C), pH, milk density (g/cm^3^), and daily milk yield (kg/day).

### 2.8 Reproductive traits

This section of the study aimed to explore the reproductive potential of Sipli sheep. The key reproductive parameters evaluated included pregnancy rates, lamb production rates, and lambing intervals in spring and summer (2021–2022), along with lamb growth rates at different ages. The study sought to determine the reproductive efficiency of the flock, and also to determine productive factors that could potentially influence reproduction. Animals were housed and managed under similar conditions and were subject to the same feeding regime. The rams were kept separately from the ewes. Five adult, healthy rams (2–3 years old) with documented breeding capabilities were selected for service. Each ram was allocated to a group of ten ewes, housed within an adjacent pen. The following traits were examined:

#### 2.8.1 Pregnancy rate.

The pregnancy rates for ewes in the spring and autumn of 2021–2022 were recorded. Reproductive efficiency was assessed based on the average number of ewes bred and the number of births that occurred in each season during the study years (2021–2022).

#### 2.8.2 Lambing interval.

The lambing interval, defined as the time elapsed between consecutive lambings for individual ewes within their respective groups, was assessed during the recording years. This data was crucial in evaluating reproductive performance and calculating the average lambing interval duration.

### 2.9 Statistical analysis

For the morphometric data of Sipli sheep, descriptive statistics were calculated, including means and the Standard Error of the Mean (SEM). Pearson’s correlation coefficient was used to assess the relationships among the morphometric traits. Statistical analysis for morphometric data was conducted using SPSS version 20.0, as described by Akbar, Javed [18]. The influence of environmental variables was analyzed using ANOVA in R software [19]. Post-hoc analysis was performed using Duncan’s Multiple Range Test (DMRT) via the agricolae package in R [20]. Four non-linear models Brody [21], Gompertz [22], Logistic [23], and von Bertalanffy [24] were employed to estimate growth rates, utilizing age and weight data with the EasyReg package in R. Model performance was compared based on the adjusted coefficient of determination (R²), Akaike’s Information Criterion (AIC), Bayesian Information Criterion (BIC), and Root Mean Square Error (RMSE). For analyses related to productive traits (wool and milk), ANOVA was conducted. Duncan’s multiple range test as modified by [25] was utilized to identify statistically significant differences between groups with more than two levels, and Tukey’s Test [26] was used to compare two groups. The mean and standard deviation of the lambing interval were calculated using SPSS. The reproductive traits also followed Tukey’s Test [26].

## 3. Results

### 3.1 Morphometric traits

#### 3.1.1 Descriptive statistics of morphometric traits.

The morphometric measurements of Sipli sheep showed clear distinctions between rams and ewes, reflecting sexual dimorphism in body size and proportions, as shown in Table 1. Rams generally exhibited larger body dimensions, with an average body length of 128.09 ± 1.43 cm and a diagonal body length of 162.82 ± 1.04 cm. In contrast, ewes displayed greater height measurements; with a height at withers of 69.82 ± 0.92 cm and a height at sacrum of 72.74 ± 0.87 cm.

**Table 1:** Morphometric measurements in ram and ewe of Sipli sheep.

Measurements	RAM N = 35		EWE N = 47	
	Mean ±SEM	CV %	Mean ±SEM	CV%
Body length (cm)	128.09 ± 1.43	11.7	123.97 ± 1.16	12.8
Diagonal body length (cm)	162.82 ± 1.04	6.7	155.5 ± 1.02	8.99
Height at wither (cm)	72.35 ± 1.02	13.5	69.82 ± 0.92	17.07
Height at sacrum (cm)	75.82 ± 1.01	13.98	72.74 ± 0.87	13
Rump length (cm)	23.43 ± 0.41	18.22	21.55 ± 0.31	19.48
Rump width (cm)	16.73 ± .032	25.1	13.8 ± 0.26	23.62
Pin width (cm)	8.45 ± 0.23	28.7	7.66 ± 0.16	29.45
Thurl length (cm)	12.25 ± 0.23	23.02	13.41 ± 0.2	32.58
Heart girth (cm)	86.88 ± 1.05	12.69	80.51 ± 0.57	9.8
Paunch girth (cm)	91.27 ± 1.15	13.2	86.98 ± 0.83	12.86
Neck length (cm)	26.69 ± 0.46	17.98	25.39 ± 0.3	16.18
Head length (cm)	25.9 ± 0.45	18.37	23.36 ± 0.19	11.3
Ear length (cm)	20.45 ± 0.93	20.01	19.99 ± 0.27	18.55
Ear width (cm)	9.94 ± 0.21	16.33	10.01 ± 0.13	17.48
Head circumference (cm)	44.79 ± 0.64	15.15	41 ± 0.53	17.73
Tail length (cm)	25.01 ± 0.41	17.47	24.1 ± 1.17	18.13
Teat length (cm)	–	–	2.8 ± 0.07	36.07
Teat diameter (cm)	–	–	3.24 ± 0.18	74.69
Testes length (cm)	16.63 ± 0.62	39.02	–	–
Testes diameter (cm)	31.46 ± 1.45	46.8	–	–
Live body weight (kg)	32.6 ± 0.5	15.95	31.55 ± 0.18	16.32

Rams showed a longer rump length (23.43 ± 0.41 cm) and a wider rump (16.73 ± 0.32 cm) compared to ewes, while ewes exhibited a longer thurl length (13.41 ± 0.2 cm). Girth measurements, specifically heart girth and paunch girth, were also greater in rams. Furthermore, rams had longer neck and head lengths. Measurements related to reproduction revealed testes measurements in rams and teat measurements in ewes. The average live body weight was comparable between the two sexes.

The detailed morphometric analysis presented in Table 2, provides a comprehensive overview of the growth trajectory in Sipli sheep lambs across different age groups. The study included a total of 45 lambs (25 males and 20 females) categorized into four age brackets: 1–3 months, 4–6 months, 7–9 months, and 10–12 months. The measurements revealed a progressive increase across the age groups for nearly all assessed parameters. Body length (BL) increased consistently from an average of 63.59 cm in the 1–3 month group to 85.23 cm in the 10–12 month group. Likewise, diagonal body length (DBL) showed a similar pattern of growth, with an increase from 73.43 cm to 111.83 cm. The height measurements at both withers (HW) and sacrum (HS) exhibited notable increases with age. Rump length and width, heart girth, and paunch girth also showed significant growth over time. Notable increases were observed in the lengths of the neck, head, ear, and tail. Head circumference increased from 27.41 cm to 45.06 cm. Notably, the average live body weight of the lambs showed a dramatic increase, progressing from 3.53 kg in the 1–3 month group to 19.30 kg in the 10–12 month group. The coefficients of variation across all parameters tended to decline with age, indicating more uniform development as the lambs matured.

**Table 2:** Morphometric measurements in lambs of different age groups of Sipli sheep.

Parameters	1-3 Months		4-6 Months		7-9 Months		10-12 Months	
	Mean ± SEM	CV%	Mean ± SEM	CV%	Mean ± SEM	CV%	Mean± SEM	CV%
Body length (cm)	63.59 ± 2.69	12.22	73.39 ± 1.6	10.18	80.64 ± 1.52	10.89	85.23 ± 0.69	8.81
Diagonal body length (cm)	73.43 ± 2.47	11.36	87.57 ± 2.97	13.39	102.66 ± 3.36	10.27	111.83 ± 1.04	9.93
Height at withers (cm)	43.16 ± 1.61	3.73	53.54 ± 1.59	2.98	63.7 ± 2.04	3.2	69.83 ± 0.5	0.71
Height at sacrum (cm)	46.29 ± 1.36	2.95	57.1 ± 1.48	2.59	66.92 ± 2.03	3.03	72.77 ± 0.5	0.68
Rump length (cm)	9.23 ± 0.28	3.06	10.44 ± 0.22	2.14	11.71 ± 0.21	1.8	13.88 ± 0.91	6.59
Rump width (cm)	11.28 ± 0.32	2.86	12.79 ± 0.3	2.31	14.04 ± 0.15	1.06	16.11 ± 0.88	5.47
Heart girth (cm)	45.95 ± 1.86	4.06	54.68 ± 1.41	2.57	61.27 ± 0.93	1.52	65.31 ± 0.53	0.82
Paunch girth (cm)	41.62 ± 2.23	5.36	51.6 ± 1.51	2.92	61.59 ± 1.99	3.24	67.66 ± 0.5	0.74
Neck length (cm)	22.94 ± 0.6	2.63	25.57 ± 0.54	2.1	27.68 ± 0.33	1.19	30.23 ± 0.79	2.62
Head length (cm)	14.5 ± 0.86	5.96	17.73 ± 0.49	2.79	20.14 ± 0.53	2.66	23.26 ± 0.65	2.78
Ear length (cm)	12.31 ± 0.64	5.24	15.63 ± 0.38	2.46	18.46 ± 0.44	2.38	21.13 ± 0.69	3.29
Ear width (cm)	9.37 ± 0.29	3.07	10.56 ± 0.24	2.25	11.44 ± 0.2	1.75	13.62 ± 0.93	6.8
Head circumference (cm)	27.41 ± 1.74	6.35	34.05 ± 1.18	3.48	40.66 ± 1.29	3.18	45.06 ± 0.57	1.27
Tail length (cm)	4.18 ± 0.27	6.47	6.59 ± 0.48	7.35	9.77 ± 0.82	8.38	13.61 ± 0.62	4.57
Teat length(cm)	0.25 ± 0.13	1.56	0.38 ± 0.16	1.21	0.46 ± 0.21	1.62	0.73 ± 0.24	2.63
Teat diameter (cm)	0.27 ± 0.12	1.12	0.41 ± 0.19	1.95	0.62 ± 0.24	1.65	0.93 ± 0.28	2.17
Testes length (cm)	4.29 ± 0.11	2.47	4.91 ± 0.13	2.72	5.43 ± 0.1	1.82	7.33 ± 0.97	3.31
Testes diameter (cm)	2.35 ± 0.05	2.33	2.62 ± 0.04	1.7	2.9 ± 0.05	1.81	4.74 ± 0.97	2.41
Live body Weight (kg)	3.53 ± 1.11	31.35	9.27 ± 1.11	11.92	15.02 ± 1.11	7.36	19.3 ± 0.54	2.82

#### 3.1.2 Correlation analysis of morphometric traits.

The correlation analysis, detailed in Tables 3 and 4, offers insights into the relationships between different morphometric traits within the Sipli sheep population, separately for rams and ewes. In the ram group (Table 3), strong positive correlations were evident between body length (BL) and several other traits, including diagonal body length (DBL), height at withers (WH), and height at sacrum (HS), indicating a proportional increase in these dimensions with increasing body length. Height measurements (WH and HS) showed a very strong correlation. Rump length (RL) and rump width (RW) showed moderate correlations with body length and diagonal body length. Heart girth (HG) and paunch girth (PG) were strongly correlated with each other. Head circumference (HC) was strongly linked to head length (HL). In the ewe group (Table 4), similar trends were observed, with body length (BL) showing strong positive correlations with diagonal body length (DBL), height at withers (HW), and rump length (RL). Rump width (RW) showed strong correlations with RL and BL.

**Table 3:** Correlation coefficients among morphometric traits of the ram group of Sipli sheep.

Traits	BL	DBL	WH	HS	RL	RW	PW	ThL	HG	PG	NL	HL	EL	EW	HC	TL	LBW
BL	1.00																
DBL	0.814**	1.00															
WH	0.774**	0.835**	1.00														
HS	0.787**	0.828**	0.993**	1.00													
RL	0.628**	0.484**	0.322**	0.347**	1.00												
RW	0.342**	0.195*	0.311**	0.311**	0.420**	1.00											
PW	0.019	0.019	0.026	0.03	0.15	0.268**	1.00										
ThL	0.076	0.007	0.178	0.204*	0.378**	0.655**	0.655**	1.00									
HG	0.768**	0.913**	0.936**	0.932**	0.360**	0.221*	0.037	0.132	1.00								
PG	0.698**	0.763**	0.752**	0.757**	0.450**	0.335**	0.186	0.073	0.857**	1.00							
NL	0.126	0.165	0.064	0.072	0.481**	0.216*	0.266**	0.008	0.033	0.153	1.00						
HL	0.434**	0.563**	0.480**	0.447**	0.283**	0.225*	0.142	0.350**	0.473**	0.325**	0.312**	1.00					
EL	0.109	0.153	0.052	0.051	0.175	0.125	0.087	0.204*	0.096	0.171	0.158	0.274**	1.00				
EW	0.097	0.216*	0.343**	0.340**	0.014	0.047	0.280**	0.023	0.208*	0.107	0.205*	0.452**	0.035	1.00			
HC	0.463**	0.559**	0.288**	0.289**	0.517**	0.300**	0.273**	0.339**	0.437**	0.454**	0.194*	0.727**	0.324**	0.035	1.00		
TL	0.210*	0.287**	0.267**	0.268**	0.320**	0.576**	0.036	0.178	0.209*	0.155	0.675**	0.456**	0.164	0.258**	0.347**	1.00	
LBW	0.698	0.752	0.857	0.171	0.107	0.325	0.454	0.153	0.763	0.155	0.45	0.335	0.186	0.073	0.757	0.11	1.00

**Correlation is significant at the 0.01 level (2-tailed).: * Correlation is significant at the 0.05 level (2-tailed).

BL: Body Length: DBL: Diagonal Body Length: WH: Height at Withers: HS: Height at Sacrum: RL: Rump Length: RW: Rump Width: PW: Pin Width: ThL: Thurl Length: HG: Heart Girth: PG: Paunch Girth: NL: Neck Length: HL: Head Length: EL: Ear Length: EW: Ear Width: HC: Head Circumference: TL: Tail Length; LBW: Live Body Weight

**Table 4:** Correlation coefficients among morphometric traits of the ewe group of Sipli sheep.

Traits	BL	DBL	HW	HS	RL	RW	PW	ThL	HG	PG	NL	HL	EL	EW	HC	TL	LBW
BL	1.00																
DBL	0.586**	1.00															
HW	0.773**	0.567**	1.00														
HS	0.133	0.10	0.205**	1.00													
RL	0.843**	0.576**	0.735**	0.194**	1.00												
RW	0.687**	0.368**	0.632**	0.12	0.787**	1.00											
PW	0.262**	0.00	0.03	−0.12	0.250**	0.371**	1.00										
ThL	0.492**	0.472**	0.610**	0.198**	0.539**	0.455**	−0.09	1.00									
HG	0.476**	0.564**	0.431**	0.153*	0.334**	0.280**	−0.06	0.429**	1.00								
PG	0.677**	0.747**	0.683**	0.148*	0.612**	0.347**	−0.03	0.573**	0.717**	1.00							
NL	0.193**	0.01	0.06	−0.07	0.09	0.179*	0.302**	0.144*	−0.01	0.04	1.00						
HL	0.188**	0.01	0.08	−0.10	0.163*	0.458**	0.441**	0.296**	0.12	0.05	0.359**	1.00					
EL	0.491**	0.332**	0.378**	0.13	0.470**	0.335**	0.229**	0.448**	0.516**	0.479**	0.442**	0.176*	1.00				
EW	0.643**	0.398**	0.533**	0.07	0.613**	0.613**	0.223**	0.638**	0.433**	0.439**	0.464**	0.470**	0.748**	1.00			
HC	0.434**	0.388**	0.598**	0.11	0.534**	0.509**	0.179*	0.509**	0.312**	0.539**	0.11	0.351**	0.321**	0.430**	1.00		
TL	0.586	0.567	0.564	0.332	0.398	0.006	0.388	0.011	0.981	0.473	0.367	0.197	0.139	0.297	0.438	1.000	
LBW	0.171	0.309	0.38	0.056	0.102	0.069	0.219	−0.074	0.373	0.081	0.172	0.171	0.309	0.38	0.056	0.016	1.00

**Correlation is significant at the 0.01 level (2-tailed).: * Correlation is significant at the 0.05 level (2-tailed).

BL: Body Length: DBL: Diagonal Body Length: WH: Height at Withers: HS: Height at Sacrum: RL: Rump Length: RW: Rump Width: PW: Pin Width: ThL: Thurl Length: HG: Heart Girth: PG: Paunch Girth: NL: Neck Length: HL: Head Length: EL: Ear Length: EW: Ear Width: HC: Head Circumference: TL: Tail Length; LBW: Live Body Weight

#### 3.1.3 Phenotypic characterization.

The phenotypic characteristics of Sipli sheep, as summarized in Table 5, illustrate the prevalence of diverse coloration patterns. Head color showed a range, with off-white (47.55%) and light brown (32.87%) being the most frequent. Eye color was predominantly black (74.36%), while eye lashes displayed greater variability, with light brown (46.39%) the most prevalent. Coat color was almost uniformly off-white (90.58%), and ear color exhibited a more balanced distribution, with dark brown (39.16%) and black (36.13%) as prominent. The nasal bridge color most often occurred as light brown (33.10%).

**Table 5:** Phenotypic characters of Sipli sheep.

Traits	Black	Brown	Dark Brown	Light Brown	Off White	White
Head Color	–	9.09%	9.56%	32.87%	47.55%	0.93%
Eyes Color	74.36%	17.48%	3.26%	4.90%	–	–
Eye Lashes	13.29%	21.91%	3.26%	46.39%	15.5%	–
Coat Color	–	–	1.24%	5.32%	90.58%	1.86%
Ear Color	36.13%	10.02%	39.16%	13.05%	1.63%	–
Nasal Bridge	17.20%	25.17%	14.69%	33.10%	10.02%	–

**Fig 2:**
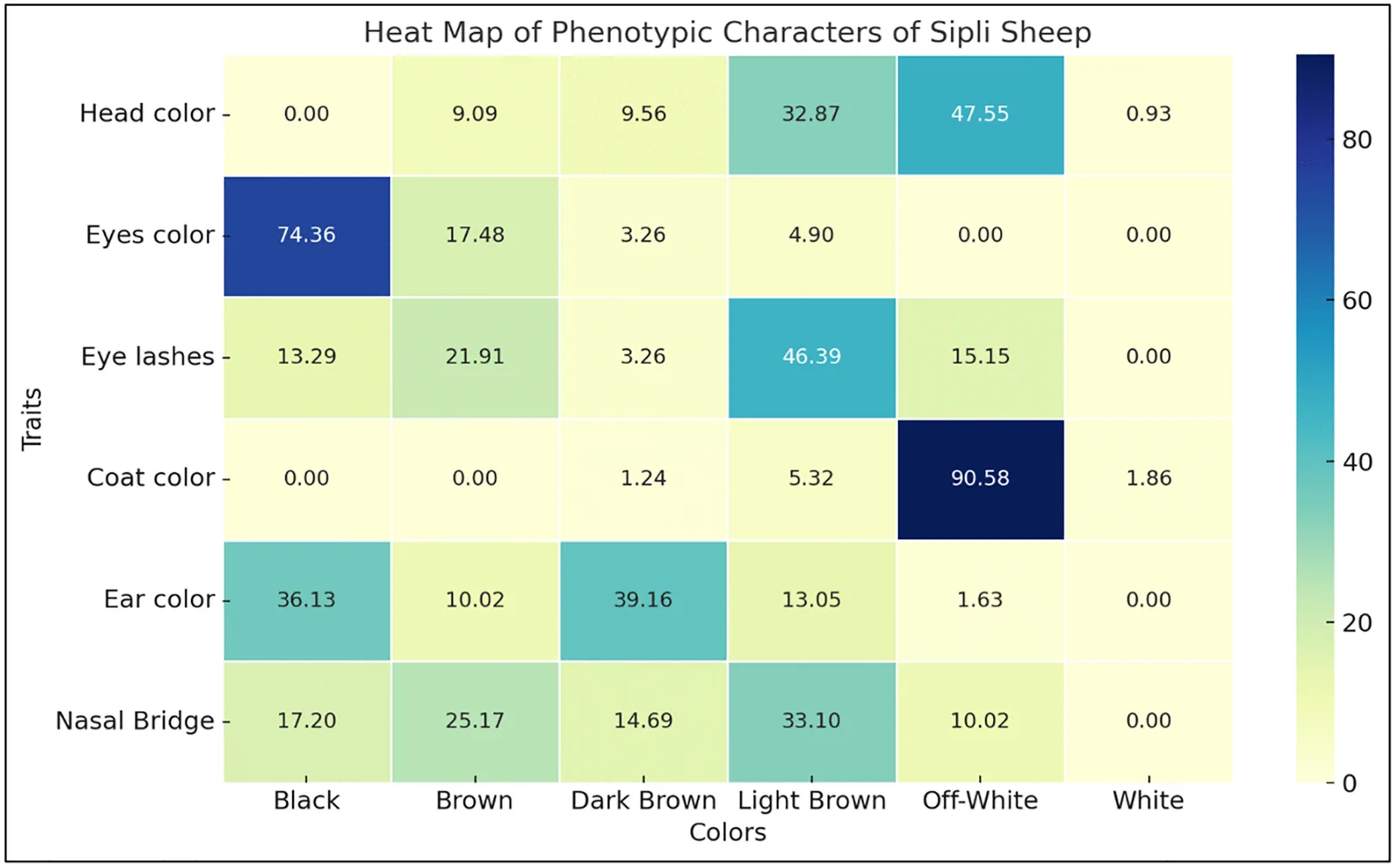
Heat map of phenotypic characteristics of Sipli sheep. These results confirm the absence of horns and a white neck color in all Sipli sheep within this study, indicating certain consistent breed-specific characteristics.

The heat map (Fig 2) provides a visual representation of the distribution of different phenotypic traits in Sipli sheep based on their color. The rows represent different phenotypic traits, including head color, eye color, eye lashes color, coat color, ear color, and nasal bridge color, while the columns represent different color categories. The intensity of the color in each cell indicates the percentage of sheep that exhibit a specific color for a given trait. Darker shades of blue represent higher percentages, indicating a more frequent occurrence of a specific color within a trait. The absence of a color indicates that no animals presented the specific colors in that traits. The results indicate a higher prevalence of black eye color, white coat color, and dark brown ear color within the Sipli sheep population.

### 3.2 Environmental factors influencing growth

The impact of environmental factors on the growth of Sipli sheep lambs was evaluated using Table 6, specifically focusing on the influence of the season and year of birth on various weight measurements. The analysis included birth weight (BWT), weight at 120 days (WT120), weight at 180 days (WT180), weight at 270 days (WT270), and weight at 365 days (WT365). The analysis revealed that the season of birth (spring vs. autumn) did not significantly (NS) impact any of the weight measurements. However, the year of birth had a significant influence on lamb growth. The year of birth significantly affected WT120, WT180, WT270, and WT365 (p < 0.001), as indicated by the highly significant p-values (***). Lambs born in 2022 exhibited higher weights at each measured interval compared to those born in 2021. The WT180 was significantly higher in lambs born in 2022 compared to 2021.

**Table 6:** Effect of season and year of birth on growth of Sipli sheep lambs.

Factors	BWT (kg)		WT120 (kg)		WT180 (kg)		WT270 (kg)		WT365 (kg)	
	N	LSM ± SEM	N	LSM ± SEM	N	LSM ± SEM	N	LSM ± SEM	N	LSM ± SEM
**Season of Birth**		**NS**		**NS**		**NS**		**NS**		**NS**
Spring	80	1.78 ± 0.29	74	9.59 ± 0.36	71	13.57 ± 0.41	66	21.27 ± 0.49	64	26.29 ± 0.52
Autumn	39	2.07 ± 0.48	37	11.72 ± 0.73	29	15.22 ± 0.82	28	18.44 ± 0.89	28	21.05 ± 0.89
**Year of Birth**		**NS**		***		***		***		**
2021	45	1.5 ± 0.41	42	10.07^b^ ± 0.60	40	14.56^a^ ± 0.74	38	20.35^b^ ± 0.85	37	24.13^b^ ± 0.89
2022	74	2.20 ± 0.30	69	11.86^a^ ± 0.38	60	16.45^b^ ± 0.43	56	21.89^a^ ± 0.51	55	25.90^a^ ± 0.56

BWT: birth weight: WT120: weight at 120 days of age: WT180: weight at 180 days of age: WT270: weight at 270 days of age: WT365: weight at 365 days of age: LSM: least squared mean: SEM: standard error mean: Spring: March-September: Autumn: October-February: N: number of animals: NS: Non-significant (p > 0.05): *** (p < 0.001): different superscripts within a column under a factor represents significant differences among different levels (p < 0.05)

#### 3.2.1 Effect of sex and birth type on lamb growth.

Table 7 showed the effect of both sex and birth type on the growth performance of Sipli sheep, analyzing birth weight (BWT) and weights at 120 (WT120), 180 (WT180), 270 (WT270), and 365 (WT365) days of age. The data demonstrated significant impacts from both factors. Male lambs consistently showed higher weights across all growth stages. For instance, male lambs presented significantly higher birth weight (2.06 kg) compared to females. By 365 days, males weighed significantly more, with an average of 28.84 kg, in contrast to the 23.71 kg average for females. The birth type also significantly impacted growth, with single-born lambs showing better growth performance than twins. This trend continued through the growth stages, with single-born lambs consistently outperforming twin-born lambs in weight gain.

**Table 7:** Effect of gender and birth type on growth of Sipli sheep lambs.

Factors	BWT (kg)		WT120 (kg)		WT180 (kg)		WT270 (kg)		WT365 (kg)	
	N	LSM ± SEM	N	LSM ± SEM	N	LSM ± SEM	N	LSM ± SEM	N	LSM ± SEM
**Gender**		**		**		**		**		**
Male	63	2.06^a^ ± 0.11	60	12.70^a^ ± 0.37	54	17.61^a^ ± 0.63	50	23.90^a^ ± 0.72	48	28.84^a^ ± 1.10
Female	56	1.76^b^ ± 0.12	51	10.98^b^ ± 0.38	46	14.92^b^ ± 0.58	44	21.12^b^ ± 0.63	44	23.71^b^ ± 0.97
**Birth Type**		***		***		***		***		***
Single	102	2.59^a^ ± 0.08	98	11.03^a^ ± 0.32	90	16.66^a^ ± 0.38	86	21.73^a^ ± 0.40	84	27.39^a^ ± 0.43
Twin	17	1.91^b^ ± 0.44	13	10.50^b^ ± 0.87	10	14.65^b^ ± 0.94	8	20.01^b^ ± 1.31	8	24.06^b^ ± 1.41

BWT: birth weight: WT120: weight at 120 days of age: WT180: weight at 180 days of age: WT270: weight at 270 days of age: WT365: weight at 365 days of age: Gender: Male, Female: Type of Birth: Single, Twin: LSM: least squared mean: SEM: standard error mean: N: number of animals: NS: Non-significant (p > 0.05): *** (p < 0.001): ** (p < 0.005): different superscripts within a column under a factor represents significant differences among different levels (p < 0.05).

### 3.3 Growth curve analysis

Table 8 presents the fit statistics for various nonlinear growth models used to describe the growth patterns in Sipli sheep. Four models were compared: Brody, Von Bertalanffy, Gompertz, and Logistic, across different categories. The fit of each model was assessed using adjusted R-squared (R²adj), Akaike Information Criterion (AIC), Bayesian Information Criterion (BIC), and Root Mean Square Error (RMSE).

**Table 8:** Fit statistics for nonlinear growth models of Sipli sheep lambs.

Category	N	Fit Statistics	Brody	Von Bertalanffy	Gompertz	Logistic
ALL	75	R2adj	0.6267	0.6236	0.6226	0.6177
		AIC	9985.476	10001.66	10009.267	10030.963
		BIC	10001.96	10018.14	10025.756	10047.405
		RMSE	14.941	15.063	15.126	15.309
**Gender**						
Female	39	R2adj	0.6302	0.6262	0.6252	0.6201
		AIC	5042.323	5046.155	5055.861	5068.381
		BIC	5056.857	5060.686	5070.394	5082.915
		RMSE	12.563	12.674	12.732	12.903
Male	36	R2adj	0.6471	0.6441	0.6421	0.6381
		AIC	4853.817	4861.681	4864.689	4875.035
		BIC	4868.102	4875.964	4878.973	4889.319
		RMSE	16.096	16.230	16.299	16.496
**Type of Birth**						
Single	40	R2adj	0.6232	0.6202	0.6182	0.6132
		AIC	5176.269	5184.465	5188.721	5201.065
		BIC	5190.876	5199.072	5203.328	5215.672
		RMSE	12.688	12.796	12.853	13.019
Twin	35	R2adj	0.6611	0.6572	0.6562	0.6511
		AIC	4689.443	4696.916	4700.732	4711.540
		BIC	4703.643	4711.116	4714.932	4725.740
		RMSE	15.538	15.677	15.748	15.952

For the overall lamb population, the Brody model demonstrated the best fit, with the highest R²adj (0.6267) and the lowest AIC (9985.476) and RMSE (14.941) values. In female lambs, the Brody model was also the best fit. In the male lambs, Brody model has highest value and for single and twin lambs also. The Logistic model showed the lowest fit across all categories, indicated by higher AIC, BIC, and RMSE values.

#### 3.3.1 Seasonal and yearly variations in growth models.

Table 9 details the performance of nonlinear growth models (Brody, Von Bertalanffy, Gompertz, and Logistic) in describing the growth trajectories of lambs, classified by season and year of birth. The analysis used adjusted R-squared (R²adj), Akaike Information Criterion (AIC), Bayesian Information Criterion (BIC), and Root Mean Square Error (RMSE) as fit statistics.

**Table 9:** Fit statistics for nonlinear growth models of lambs born in different years and seasons.

Category	N	Fit Statistics	Brody	Von Bertalanffy	Gompertz	Logistic
**Season of Birth**						
Autumn	35	R^2^_adj_	0.699	0.697	0.696	0.693
		AIC	4319.489	4325.474	4328.132	4335.845
		BIC	4333.692	4339.678	4342.335	4350.043
		RMSE	9.940	10.001	10.033	10.125
Spring	40	R^2^_adj_	0.540	0.538	0.537	0.534
		AIC	5594.478	5597.792	5599.731	5605.747
		BIC	5609.081	5612.395	5614.334	5620.350
		RMSE	9.736	9.804	9.844	9.972
**Year of Birth**						
2021	28	R^2^_adj_	0.592	0.591	0.590	0.588
		AIC	3762.02	3736.685	3764.632	3767.679
		BIC	3775.54	3777.206	3778.153	3781.214
		RMSE	16.016	16.054	16.077	16.150
2022	47	R^2^_adj_	0.615	0.613	0.611	0.608
		AIC	6234.775	6241.514	6245.141	6255.998
		BIC	6249.871	6256.609	6260.236	6271.093
		RMSE	14.425	14.512	14.558	14.699

For lambs born in autumn, the Brody model provided the best fit, with an R²adj of 0.699, the lowest AIC (4319.489), and BIC (4333.692). Conversely, in the spring, the Brody model demonstrated a slightly lower, though still acceptable fit, with an R²adj of 0.540. In terms of the year of birth, the Brody model exhibited a moderate fit for 2021-born lambs with an R²adj of 0.592 and an RMSE of 16.016. The Brody model performed slightly better for 2022-born lambs, with an R²adj of 0.615 and a lower RMSE value, 14.425.

#### 3.3.2 Parameter estimates for growth models.

Table 10 presents the parameter estimates for the Brody, Von Bertalanffy, Gompertz and Logistic growth models. These parameters define the growth characteristics of Sipli sheep lambs, classified by category (all lambs, gender, and birth type). The parameters include A (asymptotic weight), B (initial growth rate), and K (maturing rate).

**Table 10:** Estimated parameters for nonlinear growth models of Sipli sheep lambs.

Category	Parameter	Model Brody	Von Bertalanffy	Gompertz	Logistic
**ALL**	A	29.3221 ± 2.122	24.4320 ± 1.000	23.3182 ± 0.801	21.5701±0.525
	B	0.8831 ± 0.008	0.4534 ± 0.009	1.7091 ± 0.042	3.5420±0.154
	K	0.0033 ± 0.0001	0.0064 ± 0.001	0.0073 ± 0.001	0.0120±0.001
**Gender**					
Female	A	25.5071 ± 2.063	21.9964 ±1.070	21.1476 ±0.876	19.7710±0.594
	B	0.8701 ± 0.011	0.4410 ± 0.012	1.6533 ±0.057	3.3451±0.202
	K	0.0045 ± 0.001	0.007 ± 0.001	0.008 ± 0.001	0.012±0.007
Male	A	34.2821 ± 4.292	27.0346 ± 1.793	25.8330 ± 1.404	23.5910±0.886
	B	0.8965 ± 0.021	0.4664 ± 0.012	1.7683 ± 0.059	3.7576±0.225
	K	0.0032 ± 0.002	0.0052 ± 0.002	0.0071 ± 0.001	0.0112±0.0012
**Type of Birth**					
Single	A	25.5260 ±2.121	21.950 ± 1.092	21.0870±0.893	19.6921±0.603
	B	0.8694 ±0.011	0.4412 ± 0.012	1.6493 ± 0.056	3.3320±0.200
	K	0.0043 ± 0.001	0.0067 ± 0.001	0.0081 ± 0.001	0.0123±0.001
Twin	A	34.3721 ±4.142	27.4680 ±1.746	26.0080 ±1.369	23.7812±0.867
	B	0.8967 ±0.011	0.4661 ± 0.012	1.7731 ± 0.058	3.7740±0.224
	K	0.0031 ± 0.001	0.0062 ± 0.001	0.0077 ±0.001	0.0112±0.001

The Brody model showed the highest asymptotic weight (A = 29.3221 ± 2.122 kg) for all lambs. In contrast, the Logistic model had the lowest (A = 21.5701 ± 0.525 kg). The initial growth rate (B) was highest in the Logistic model (B = 3.5420 ± 0.154), while the Brody model had the lowest (B = 0.8831 ± 0.008). The maturing rate (K) was highest in the Logistic model (K = 0.0120 ± 0.001) and lowest in Brody (K = 0.0033 ± 0.0001). Regarding gender, the asymptotic weight was significantly higher in male lambs (A = 34.2821 ± 4.292) than in female lambs (A = 25.5071 ± 2.063), indicating a larger size potential for males. For birth types, twin-born sheep have a larger estimated asymptotic weight (A = 34.3721 ± 4.142).

#### 3.3.3 Seasonal and yearly variations in growth models.

Table 11 presents the estimated parameters for various nonlinear growth models (Brody, Von Bertalanffy, Gompertz, and Logistic), applied to the Sipli sheep lambs born in different seasons and years. The study focuses on the asymptotic weight (A), initial growth rate (B), and maturing rate (K).

**Table 11:** Estimated parameters for lambs born in different years and seasons.

Category	Parameter	Model Brody	Von Bertalanffy	Gompertz	Logistic
**Season of Birth**					
Autumn	A	47.0612 ± 12.208	30.2520 ± 2.972	27.6081 ± 2.117	23.9380 ± 1.159
	B	0.9051 ± 0.021	0.4580 ± 0.013	1.7172 ± 0.057	3.5033 ± 0.179
	K	0.0013 ± 0.001	0.0041 ± 0.001	0.0054 ± 0.001	0.0095 ± 0.001
Spring	A	29.0401 ± 3.328	24.3862 ± 1.572	23.2947 ± 1.254	21.5562 ± 0.809
	B	0.8561 ± 0.014	0.4274 ± 0.013	1.5855 ± 0.061	3.1260 ± 0.208
	K	0.0032 ± 0.001	0.0060 ± 0.001	0.0072 ± 0.001	0.0112 ± 0.001
**Year of Birth**					
2021	A	52.9244 ± 23.897	30.5702 ± 4.444	27.5621 ± 3.050	23.5801 ± 1.584
	B	0.9213 ± 0.030	0.4731 ± 0.019	1.7862 ± 0.082	3.7485 ± 0.269
	K	0.0012 ± 0.001	0.0044 ± 0.001	0.0056 ± 0.001	0.0092 ± 0.001
2022	A	29.4214 ± 2.739	24.7845 ± 1.321	23.6832 ± 1.060	21.9176 ± 0.691
	B	0.8510 ± 0.011	0.4213 ± 0.010	1.5594 ± 0.046	3.0350 ± 0.155
	K	0.0033 ± 0.001	0.0064 ± 0.001	0.0071 ± 0.001	0.0112 ± 0.001

For autumn-born lambs, the Brody model estimated the highest asymptotic weight (A = 47.0612 ± 12.208). The Logistic model consistently showed the lowest asymptotic weight. In spring-born lambs, the Brody model exhibited a lower asymptotic weight (A = 29.0401 ± 3.328). Regarding the year of birth, lambs born in 2021, the Brody model estimated a notably higher asymptotic weight (A = 52.9244 ± 23.897). The Logistic model again showed the highest initial growth rate and maturing rate.

#### 3.3.4 Growth curves in Sipli sheep.

**Fig 3:**
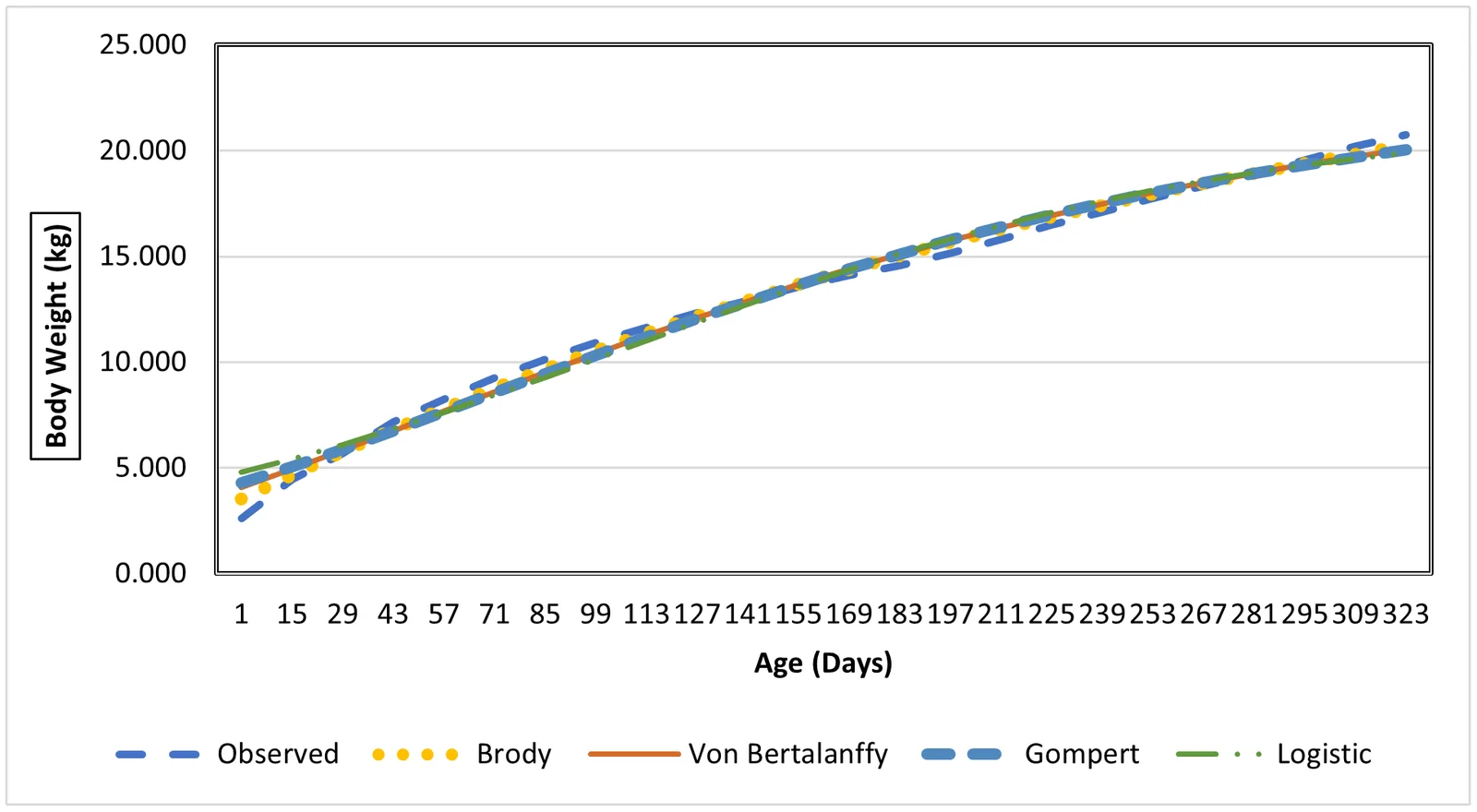
Body weights observed and predicted by different nonlinear models for Sipli lambs.

The growth curves generated by four nonlinear models (Brody, Von Bertalanffy, Gompertz and Logistic) effectively capture the growth dynamics of Sipli lambs. The observed body weights, depicted by the blue dashed line, show a consistent increase over time. Each model’s predictions, represented by different colored lines, closely mirrored the observed trend. The Brody (yellow dotted line) and Von Bertalanffy (orange solid line) models provided the closest fit, particularly during the early growth phase. The Gompertz (blue dashed line) and Logistic (green dashed line) models also followed the general growth trend. As the lambs aged, the predicted weights from all models converged, indicating consistent growth patterns (Fig 3).

#### 3.3.5 Gender-specific growth curves.

**Fig 4:**
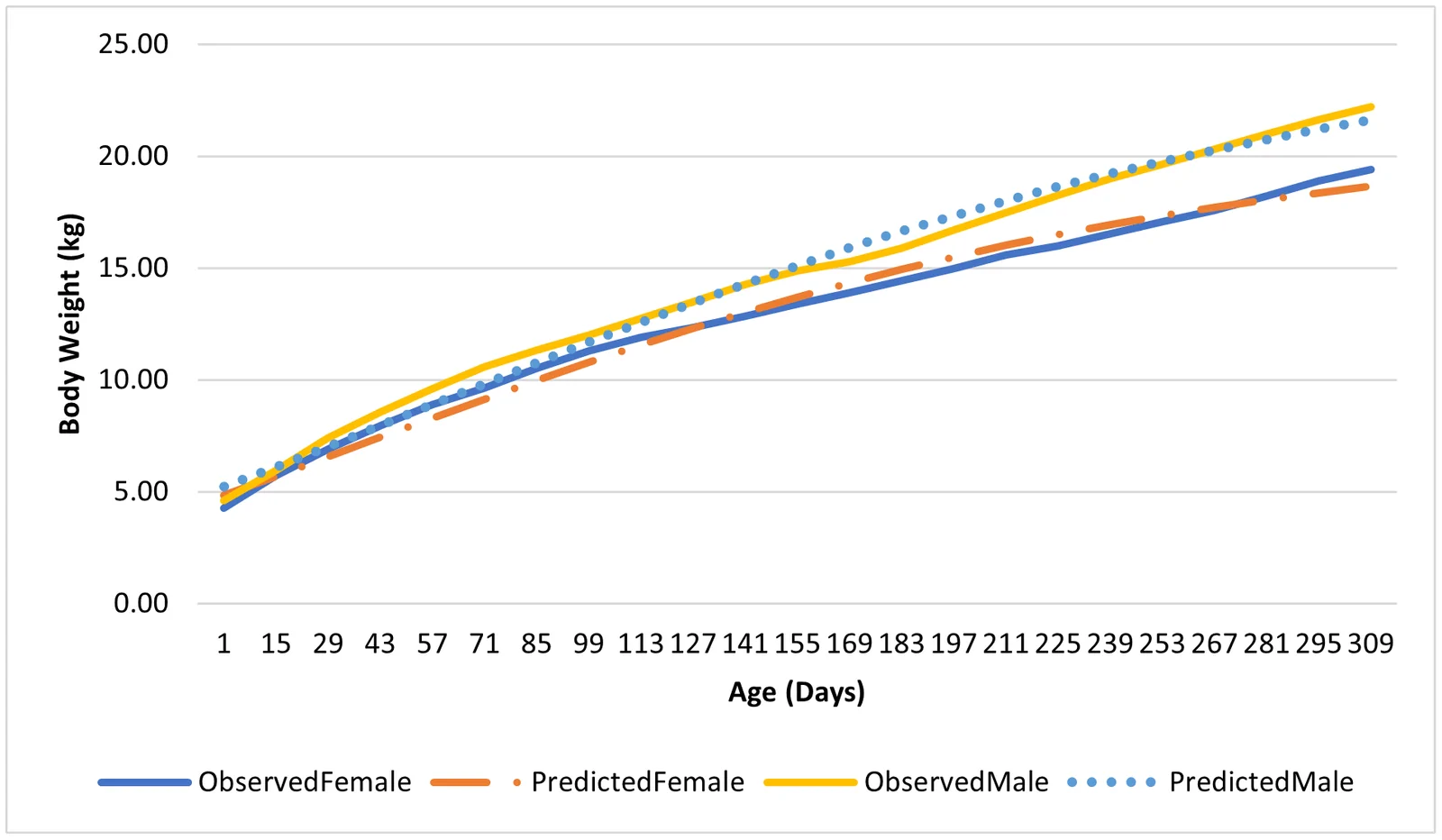
Observed and predicted body weights of male and female Sipli lambs.

The Brody model effectively describes the growth of Sipli lambs, showing distinct growth trajectories for males and females. The observed body weights of female lambs (blue solid line) increase from approximately 5 kg to around 17 kg over 309 days. The Brody model’s predictions for females (orange dashed line) closely follow the observed data. Male lambs (yellow solid line) exhibit higher body weights, increasing from about 6 kg to over 20 kg. The Brody model’s predictions for males (blue dotted line) accurately reflect the faster growth rate. The figure highlights that the Brody model accurately captured the growth dynamics and provided effective predictions for both male and female lambs over the specified period (Fig 4).

#### 3.3.6 Growth curves in single and twin lambs.

**Fig 5:**
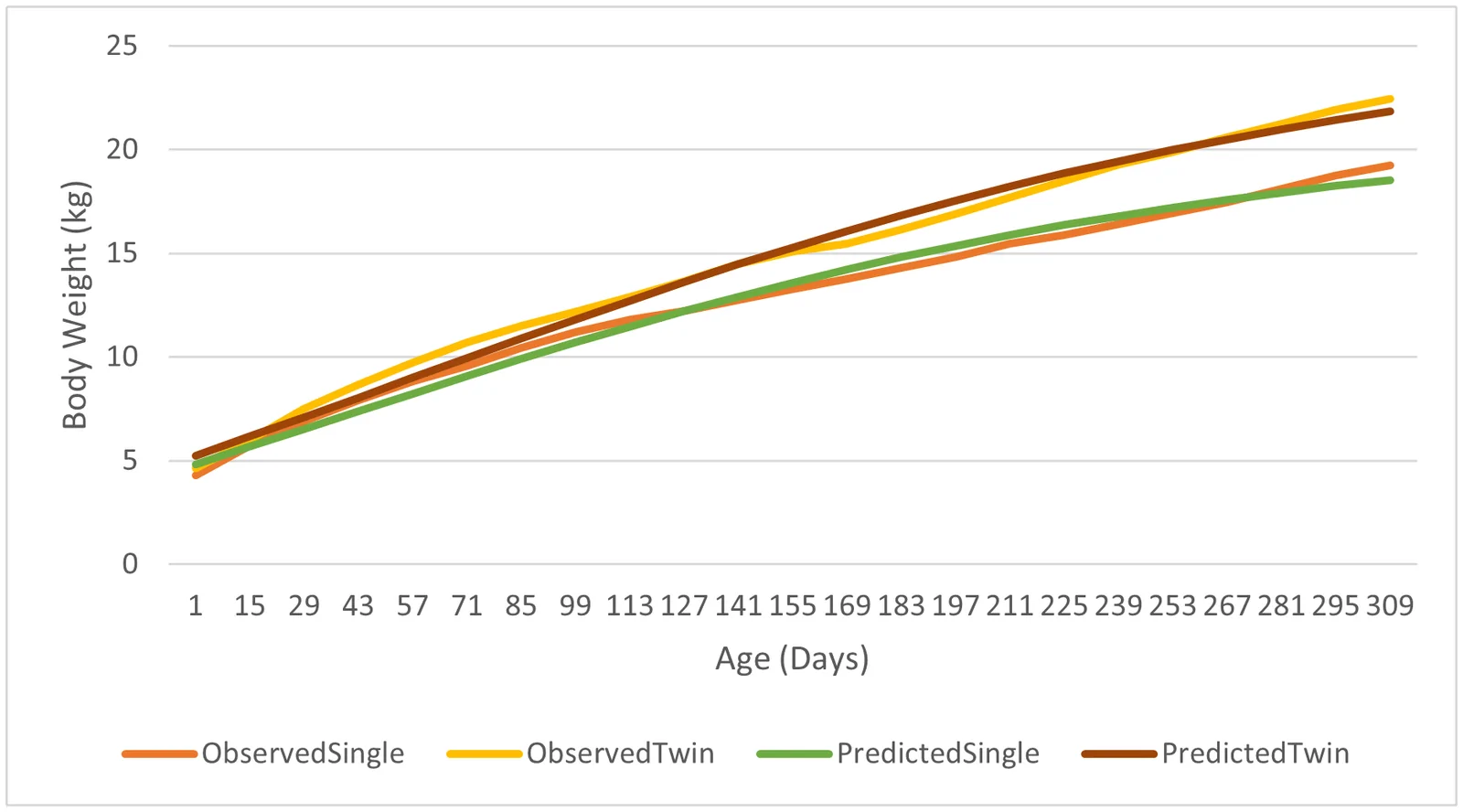
Body weights observed and predicted by the brody model for single and twin Sipli lambs.

The growth patterns of single and twin Sipli lambs, described using the Brody model, are presented in Fig 5. The observed weights of single lambs (yellow solid line) began at about 5 kg and reached roughly 19 kg by day 309. The model’s predictions for single lambs (brown solid line) closely followed the observed growth. Twin lambs exhibited a slightly higher starting weight and a faster growth rate (orange solid line), approaching 18 kg by the end of the study. The model’s predictions for twin lambs (dark yellow solid line) similarly mirrored the observed trend.

### 3.4 Productive traits

#### 3.4.1 Wool quality and quantity traits.

The analysis of wool traits in Sipli sheep, presented in Table 12, revealed the effects of gender, age, season of shearing, and season of birth on wool quality. The parameters assessed include heterotypical fiber (%), medullated fiber (%), true fiber (%), fiber diameter (µm), fiber length (cm), staple length (cm), and wool weight (kg). Significant variations (P < 0.05) in several wool traits were observed based on the factors analyzed. Rams displayed a higher medullated fiber content (19.27%), a thicker fiber diameter (33.16 µm), longer staple length (7.07 cm), and heavier wool yield (2.5 kg) compared to ewes. Wool traits changed with the age of the sheep. Older sheep (3 and 4 years old) produced significantly more wool (3.03 and 3.0 kg, respectively) and a thicker fiber diameter than the younger animals. Wool sheared in autumn had a significantly thicker fiber diameter (34.94 µm) and longer fiber length (8.26 cm) than wool sheared in spring. Sheep born in winter had lower heterotypical fiber content (35.5%). Furthermore, the highest wool yield was observed for those born in spring (2.58 kg).

**Table 12:** Least squares means for various factors affecting wool trait quality.

Overall Mean	Trait	N	Heterotypical fiber (%)	Medullated fiber (%)	True fiber (%)	Fiber diameter (µm)	Fiber length(cm)	Staple length(cm)	Wool weight (kg)
Gender	Ewe	44	42.65^a^ ± 15.36	12.56^a^ ± 8.61	44.31^a^ ± 10.68	33.41^a^ ± 0.17	7.67^a^ ± 1.49	6.36^a^ ± 1.15	1.9^a^ ± 0.95
	Ram	22	41.36^a^ ± 14.62	19.27^b^ ± 8.64	39.54^a^ ± 10.04	33.16^b^ ± 0.16	7.96^a^ ± 1.44	7.07^b^ ± 1.05	2.5^b^ ± 1.00
Age of animal	1Year	27	46.59^a^ ± 14.59	13.51^a^ ± 9.46	39^b^ ± 8.31	30.76^a^ ± 0.03	7.53^a^ ± 1.86	6.39^a^ ± 1.29	1.2^b^ ± 0.39
	2Year	7	44.85^a^ ± 19.13	18.28^a^ ± 13.06	36^ab^ ± 8.95	31.40^b^ ± 0.03	7.67^a^ ± 1.86	6.6^a^ ± 1.43	1.34^b^ ± 0.29
	3Year	16	37.68^a^ ± 14.37	15.06^a^ ± 8.53	47.37^a^ ± 10.42	31.88^c^ ± 0.03	8.36^a^ ± 0.89	7.05^a^ ± 1.01	3.03^a^ ± 0.62
	4Year	16	38.25^a^ ± 13.38	15.18^a^ ± 7.49	46.93^ab^ ± 12.14	32.22^d^ ± 0.03	7.61^a^ ± 0.85	6.49^a^ ± 0.90	3.0^a^ ± 0.41
Season of shearing	Autumn	33	41.87^a^ ± 15.21	14^a^ ± 9.48	43.88^a^ ± 9.29	34.94^a^ ± 0.25	8.26^a^ ± 1.48	6.92^a^ ± 1.17	2.08^a^ ± 1.06
	Spring	33	42.57^a^ ± 15.06	15.6^a^ ± 8.83	41.5^a^ ± 11.87	31.68^b^ ± 0.12	7.26^b^ ± 1.30	6.27^b^ ± 1.07	2.11^a^ ± 0.95
Season of birth	Spring	30	47.7^ab^ ± 13.65	13.73^ab^ ± 9.14	38.16^b^ ± 7.83	32.45^a^ ± 2.23	7.69^a^ ± 1.14	6.6^a^ ± 1.02	2.58^b^ ± 0.95
	Winter	12	35.5^b^ ± 13.44	21.75^b^ ± 9.54	42.75^a^ ± 8.12	30.76^a^ ± 2.26	7.6^a^ ± 1.93	6.54^a^ ± 1.46	1.25^a^ ± 0.25
	Autumn	18	35.83^ab^ ± 14.07	12.83^a^ ± 7.4	51.66^ab^ ± 1.4	31.75^a^ ± 2.28	8.32^a^ ± 1.70	6.92^a^ ± 1.23	2.27^b^ ± 0.88
	Summer	6	47.5^a^ ± 17.68	12.16^b^ ± 8.18	38.66^b^ ± 9.83	31.35^a^ ± 2.12	6.78^a^ ± 0.36	5.68^a^ ± 0.41	0.86^a^ ± 0.19

Different superscript letters indicate significant differences (P < 0.05): shared letters mean no significant difference. Gender, Male and Female: Season of Birth: Spring (March-May), Winter (December- February), Autumn (October-November), Summer (June-September): Season of Shearing: Autumn (October to February) and Spring (March to September) and age of the animal: 1 year, 2 years, 3 years and older than 3 years

#### 3.4.2 Milk yield and composition.

The analysis of milk composition across different lactation days is shown in Table 13, revealed the significant changes in the physio-chemical properties of Sipli sheep milk over the lactation period. The study analyzed parameters including salts, protein, fat, lactose, solids-not-fat (SNF), freezing point, milk sample temperature, pH, density, and daily milk yield.

**Table 13:** Physio-chemical milk composition of milk traits at different lactation days.

Lactation Day	Salts%	Proteins%	Fat%	Lactose%	SNF%	Freezing. Point° C	Milk sample Temperature °C	pH	Density/cm³	Milk yield kg/Day
5	0.59^g^ ± 0.01	4.81^a^ ± 0.12	5.20^e^ ± 0.15	5.36^a^ ± 0.43	9.15^g^ ± 0.10	−0.543^c^ ± 0.02	34.95^a^ ± 2.67	6.30^g^ ± 0.09	1.02^a^ ± 0.00	0.45^b^ ± 0.10
20	0.61^f^ ± 0.01	4.65^ab^ ± 0.12	5.45^d^ 0.16	5.29^ab^ ± 0.23	9.35^f^ ± 0.10	−0.53^bc^ ± 0.02	33.23^a^ ± 2.32	6.35^fg^ ± 0.09	1.05^a^ ± 0.07	0.50^b^ ± 0.10
35	0.63^e^ ± 0.01	4.49^bc^ ± 0.11	5.65 cd ± 0.12	5.12^bc^ ± 0.21	9.52^e^ ± 0.18	−0.53^abc^ ± 0.02	33.68^a^ ± 2.68	6.45^ef^ ± 0.10	1.03^a^ ± 0.00	0.75^a^ ± 0.10
50	0.64^de^ ± 0.01	4.35 cd ± 0.13	5.82^bc^ ± 0.12	4.96 cd ± 0.21	9.65^de^ ± 0.10	−0.52^abc^ ± 0.02	35.45^a^ ± 2.50	6.47^de^ ± 0.08	1.03^a^ ± 0.00	0.85^a^ ± 0.10
65	0.65 cd ± 0.01	4.20^de^ ± 0.14	5.99^ab^ ± 0.12	4.84^de^ ± 0.19	9.75 cd ± 0.10	−0.51^abc^ ± 0.02	36.3^a^ ± 80.50	6.58 cd ± 0.07	1.03^a^ ± 0.00	0.45^b^ ± 0.10
80	0.66^bc^ ± 0.01	4.05^ef^ ± 0.16	6.17^a^ ± 0.14	4.67^ef^ ± 0.18	9.85^bc^ ± 0.10	−0.50^ab^ ± 0.02	34.86^a^ ± 2.52	6.62^bc^ ± 0.09	1.05^a^ ± 0.08	0.40^b^ ± 0.10

**Fig 6:**
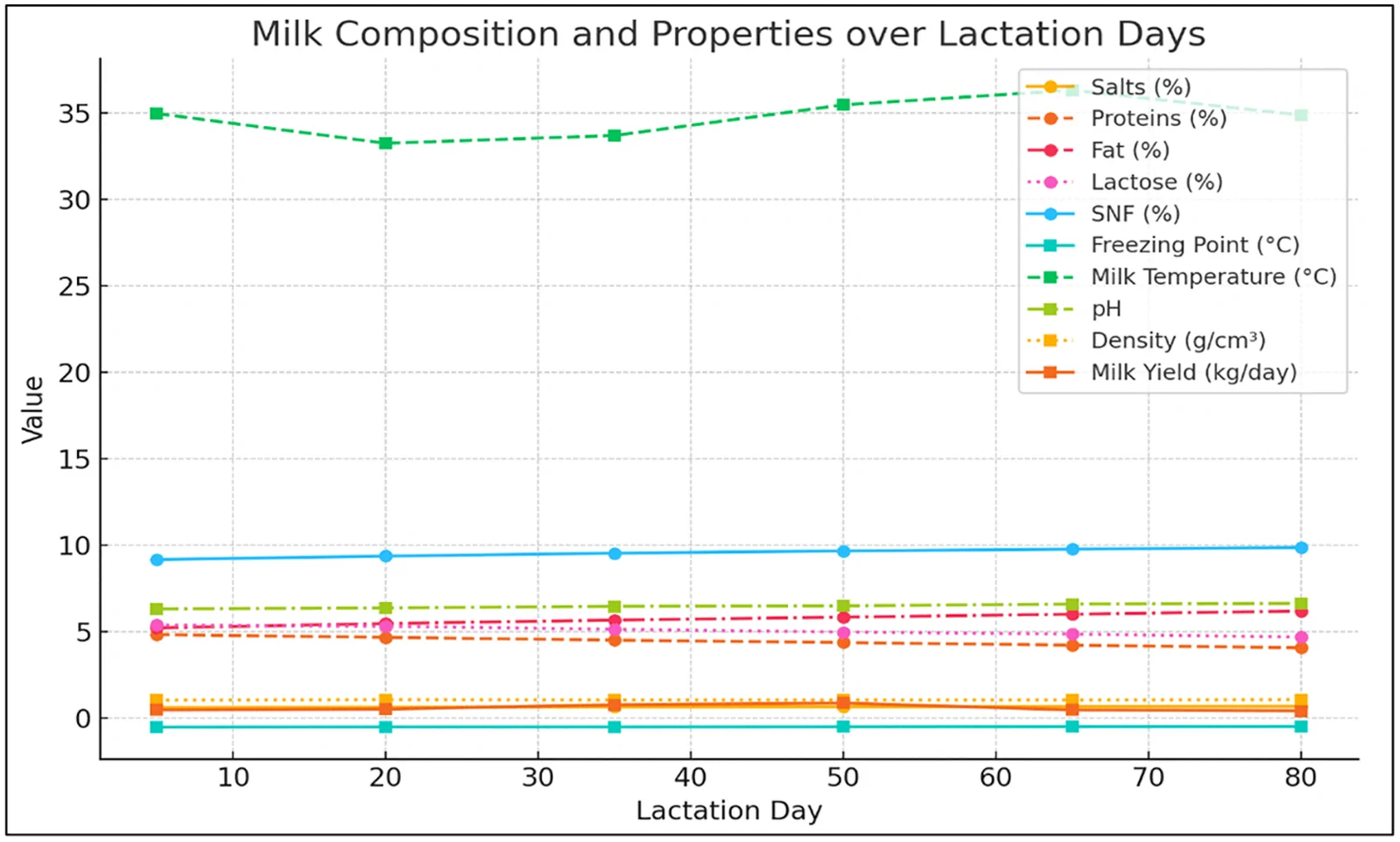
Physio-chemical milk composition of milk traits at different lactation days.

The changes in physio-chemical milk composition and properties over different lactation days is presented in Fig 6. The milk temperature (green dashed line) varied, while protein content (orange dashed line), lactose content (pink dotted line), and milk yield (orange solid line) initially increased. The SNF (cyan solid line), freezing point (teal solid line), pH (green dashed line) and density (yellow dotted line) showed minimal changes over the course of the study. The milk yield (orange solid line) starts at 0.45 kg/day at 5 days and increases in peak on day 50 and declined in 80 days.

### 3.5 Reproductive traits

The reproductive traits of Sipli sheep were assessed across the spring and autumn seasons of 2021 and 2022, and the findings are summarized in Table 14. Key metrics included pregnancy rate, birth type (single or twin), and lambing rate. The pregnancy rates varied substantially across seasons and years. In 2021, the pregnancy rate was higher in spring (78%) compared to autumn (53.06%). The pattern reversed in 2022, with a high pregnancy rate in spring (95.83%) and a significant decline in autumn (40.43%). The percentage of single births showed consistency across seasons and years, with the majority of births being single. The lambing rates demonstrated the productivity of the ewes with spring seasons showing better reproductive outcomes in both years, with higher pregnancy rates and lambing rates compared to autumn.

**Table 14:** Reproductive traits of Sipli sheep 2021-2022.

Traits	2021						2022					
	Spring	%	Autumn	%	T-Value	P-Value	Autumn	%	Spring	%	T-Value	P-Value
No. Of Sheep	50		49				47		48			
**Pregnancy Rate**												
Pregnant Sheep	39	78%	26	54%	1.15	0.009	19	40%	46	96%	2.45	0.019
Non-Pregnant Sheep	11	22%	23	47%	2.18	0.025	28	59%	2	4%	1.58	0.025
**Birth Type**												
Single Birth	36	86%	24	86%	1.03	0.008	19	83%	56	85%	0.87	0.007
Twin Birth	6	14%	4	14%	1.47	0.002	4	17%	10	15%	1.29	0.002
Total. Lamb Born	42		28		2.14	0.009	23		66		2.9	0.009
Lambing Rate	110.5263		107.6923		1.98	0.002	0.003	121.0526		143.4783	3.12	0.003

### 3.6 Discussion

The results of this study provides valuable insights into the morphometric characteristics and the productive and reproductive performance of the Sipli sheep breed in Punjab, Pakistan. These findings are essential for developing effective breeding strategies, conservation efforts, and management practices. The observed morphometric measurements revealed significant sexual dimorphism, with rams generally larger than ewes. These findings align with studies in other sheep breeds, where males typically exhibit larger body dimensions [27,28]. The correlation analysis highlighted strong relationships between various body measurements, such as body length, heart girth, and body weight, particularly in the ram group. This supports the findings of Akbar, Javed (18) and Arsalan, Ullah (29) who also identified heart girth as a critical measurement for estimating body weight in Thalli sheep. The phenotypic characterization revealed variations in coat and head color patterns, with coat color being predominately white, and eyes being predominately black. Similar studies have explored phenotypic variations in sheep breeds, emphasizing the importance of these traits in breed identification and conservation [30]. The observed variations in wool traits in present study findings are consistent with the findings of Singh, Gahlot (15) who have investigated wool quality traits.

Our analysis of environmental factors revealed that the year of birth significantly impacted growth traits, with lambs born in 2022 showing higher weights than those born in 2021. This could be attributed to variations in environmental conditions, such as forage availability or management practices, influencing growth rates [31,32]. Moreover, our study also demonstrated the important effect of birth type on lamb growth traits, with single-born lambs showing the best growth performance, as reported by Mathapo, Mugwabana [33]. This aligns with the findings of other studies, which showed that single-born lambs have better growth performance than twin-born lambs, because of access to more resources [34]. The analysis of milk composition showed changes over lactation days, with fat and SNF content increasing as lactation progressed, which matches the findings of Yasmin, Iqbal [35]. We observed that milk yield peaked around day 50 after lambing, which is an important aspect to consider for dairy sheep breeding practices.

We used nonlinear growth models that yielded a robust framework for analyzing the growth curves of Sipli sheep. The Brody model demonstrated the best fit for overall lamb growth, aligning with the findings of Sharif, Ali (14) and [36] in Lohi and Thalli sheep, respectively. The Brody model’s effectiveness in capturing growth dynamics, especially in male lambs, underscores its potential for improving breeding strategies and management practices [37]. The analyses showed that twin lambs tended to have higher final body weights than single lambs, which is in contrast with the findings by Sharif, Ali [14].

The reproductive traits analyzed in present study showed significant variations in pregnancy and lambing rates across the years and seasons. Higher lambing rates were observed in the spring seasons. This seasonal variability is consistent with the findings of Kridli, Abdullah [38] highlighting the impact of environmental factors on reproductive performance in sheep. The findings from the current study offer a deeper understanding of Sipli sheep and their potential for improvement. The identification of strong correlations between specific morphometric traits, particularly heart girth, body length, and body weight, mirrors results in Thalli sheep [18,29] and in the Bardhoka breed [39] underscoring the significance of these measurements as reliable indicators of body mass. This is an important finding, as it simplifies the estimation of body weight, a critical factor in animal management and breeding programs [40,41]. Moreover, the strong performance of the Brody model in describing the growth curves, aligns with research on Lohi [14] and Kordi sheep [32].

Furthermore, the significant impact of the year of birth on growth traits highlights the influence of environmental factors, which have also been noted in studies in West African Dwarf sheep [42] and it emphasizes the need for accounting for these effects in breeding programs to obtain accurate genetic evaluations [43]. The advantages conferred by male sex and single births on growth confirm similar results in Deccani lambs [44] and align with general expectations in livestock production [45].

We observed variations in wool quality traits based on gender, age, season of shearing, and birth is valuable information for optimizing wool production practices. The superior wool traits observed in rams, older animals, and those born in spring or autumn, contribute towards the understanding of optimal breeding and shearing schedules, to maximize the quality and quantity of wool. This study provides valuable insights to improve the production potential of Sipli sheep for their wool and meat traits, aligning with the findings of Niaz, Kaleri [46] and Safi, Kaleri [47]. Similarly, the changes in milk composition over the lactation period inform optimal milking practices, which is critical for breeds, such as Kajli sheep, that are used for milk production [35]. The reproductive traits highlight the complexities of ewe’s reproduction. The differences in pregnancy and lambing rates across the years and seasons emphasize the influence of environmental conditions on reproductive performance [38,48]. This information will contribute towards targeted breeding programs that improve the weight gains for specific traits [49].

The current study emphasis on morphometric traits and the evaluation of growth curves contributes to the knowledge base for Sipli sheep, especially in light of the broader literature on sheep breeds. Similar to the findings of Yakubu [50] and Chitra, Rajendran [51] who identified chest circumference, body length, and heart girth as significant predictors of body weight in Uda sheep, Malabari goats, and several other breeds. This study showed that the Brody model was the best fit with the results by Hojjati and Ghavi Hossein-Zadeh [52]. This aligns with these studies and confirms the potential for a simple and effective measurement to be made for the prediction of body weight. This is particularly relevant for the management of flocks, where regular weight assessments are crucial. Additionally, the analysis of milk production traits also showed a relationship with the lactation stage. The findings from this study can also be used to help optimize the milking practices for the dairy sheep farmers of Sipli. These insights and findings are essential for management and breeding.

## Conclusion

In conclusion, this study provides a comprehensive assessment of the morphometric traits, productive, and reproductive performance of Sipli sheep in Punjab, Pakistan. The findings highlight significant sexual dimorphism in body measurements, with rams exhibiting larger overall dimensions. The morphometric data also revealed consistent growth patterns across different age groups, showing that the body length, diagonal body length, and other related dimensions increase over time. Furthermore, the study demonstrated the effect of birth type and gender on the growth. This study also showed that the Brody model was the best fit for describing the growth patterns. The examination of wool traits identified significant differences in wool quality, demonstrating that certain factors, such as the gender, age of animal and season, affect wool fiber diameter, length, and yield. Likewise, the lactation period showed variations in milk composition. The variations observed in the reproductive traits, mainly pregnancy rate and lambing rates across different years and seasons, highlight the impacts on environmental effects on reproductive performance. Overall, this research contributes valuable insights for effective breed characterization, improved management practices, and informed breeding strategies for Sipli sheep.

## References

[1] Khan NA, Shah AA, Chowdhury A, Wang L, Alotaibi BA, Muzamil MR. Rural households’ livelihood adaptation strategies in the face of changing climate: a case study from Pakistan. Heliyon. 2024;10(6):e28003. doi: 10.1016/j.heliyon.2024.e28003 38509972 PMC10951648

[2] Ahmad N, Yuan H, Zhu Z, Chu T, Liu J, Song Y. Pakistan sheep industry its constrains and future trends. Trop Anim Health Prod. 2024;56(9):399. doi: 10.1007/s11250-024-04246-x 39613911

[3] Muhammad M, Abdullah M, Javed K, Khan M, Jabbar M. Goat production systems in Punjab, Pakistan. JAPS: J Animal Plant Sci. 2015;25(3).

[4] Mazinani M, Rude B. Population, world production and quality of sheep and goat products. Am J Animal Vet Sci. 2020;15(4):291–9. doi: 10.3844/ajavsp.2020.291.299

[5] Engdawork A, Belayhun T, Aseged T. The role of reproductive technologies and cryopreservation of genetic materials in the conservation of animal genetic resources. Ecological Genetics and Genomics. 2024;31:100250. doi: 10.1016/j.egg.2024.100250

[6] Silveira RMF, McManus CM, Carrara ER, De Vecchi LB, de Sousa Carvalho JR, Costa HHA, et al. Adaptive, morphometric and productive responses of Brazilian hair lambs: Crossing between indigenous breeds - A machine learning approach. Small Ruminant Res. 2024;232:107208. doi: 10.1016/j.smallrumres.2024.107208

[7] Idris M, Farooq U, Rashid H, Lashari MH, Riaz U, Khan MA. A preliminary study on the dynamics of serum color in perspective to hemoglobin and bilirubin in indigenous sheep of Pakistan. J Exp Zool Part A: Ecol Integrative Physiol. 2024;341(2):123–9. 10.1002/jez.2765 38010902

[8] Paneru U, Moghaddar N, van der Werf J. Comparison between multiple-trait and random regression models for genetic evaluation of weight traits in Australian meat sheep. J Anim Sci. 2024;102:skae038. doi: 10.1093/jas/skae038 38334207 PMC10896620

[9] Qureshi ZI, Farid AH, Babar ME, Tanveer Hussain TH. Leptin gene polymorphism in Lohi, Kajli and Spili breeds of sheep. 2015.

[10] Faraz A, Waheed A, Ishaq H, Mirza R. Rural development by livestock extension education in southern Punjab. J Fish Livestock Prod. 2019;7(1):287.

[11] Idris M, Farooq U, Lashari M, Qayyum S, Arshad A, Riaz U, et al. Dynamics of serum biochemical attributes in indigenous Sipli sheep breed kept under intensive farming system. JAPS: J Animal Plant Sci. 2024;34(1).

[12] FAO. Phenotypic characterization of animal genetic resources: FAO Animal Production and Health Guidelines No. 11. Rome: FAO; 2012.

[13] Sharif N, Ali A, Dawood M, Khan MI-R, Do DN. Environmental effects and genetic parameters for growth traits of Lohi sheep. Animals. 2022;12(24):3590. 36552510 10.3390/ani12243590 PMC9774388

[14] Sharif N, Ali A, Mohsin I, Ahmad N. Evaluation of nonlinear models to define growth curve in Lohi sheep. Small Ruminant Research. 2021;205:106564. doi: 10.1016/j.smallrumres.2021.106564

[15] Singh H, Gahlot G, Narula H, Pannu U, Chopra A. Effect of genetic and non-genetic factors on wool traits in Magra sheep. Veterinary Practitioner. 2018;(1):119–22.

[16] Koonce KL. Mixed-model least squares and maximum likelihood computer program (LSMLMW PC-1 version). JSTOR. 1990.

[17] Dzidic A, Rovai M, Poulet JL, Leclerc M, Marnet PG. Review: milking routines and cluster detachment levels in small ruminants. Animal. 2019;13(S1):s86–93. doi: 10.1017/S1751731118003488 31280744

[18] Akbar MA, Javed K, Faraz A, Waheed A. Principal component analysis of morphometric traits explain the morphological structure of Thalli sheep. 2021.

[19] Lenth RV. Least-squares means: theRPackagelsmeans. J Stat Soft. 2016;69(1). doi: 10.18637/jss.v069.i01

[20] de Mendiburu F, de Mendiburu MF. Package ‘agricolae’. R Package. 2019. 1143–9.

[21] Brody S, Lardy HA. Bioenergetics and growth. J Phys Chem. 1946;50(2):168–9. doi: 10.1021/j150446a008

[22] Laird AK. Dynamics of relative growth. 1965. 5865687

[23] Nelder JA. The fitting of a generalization of the logistic curve. Biometrics. 1961;17(1):89. doi: 10.2307/2527498

[24] Von Bertalanffy L. Quantitative laws in metabolism and growth. Q Rev Biol. 1957;32(3):217–31. doi: 10.1086/401873 13485376

[25] Kramer CY. Extension of multiple range tests to group correlated adjusted means. Biometrics. 1957;13(1):13. doi: 10.2307/3001898

[26] Keselman HJ, Rogan JC. The Tukey multiple comparison test: 1953–1976. Psychological Bulletin. 1977;84(5):1050–6. doi: 10.1037/0033-2909.84.5.1050

[27] Haque M, Sarder M, Islam M, Khaton R, Islam M, Hashem M. Morphometric characterization of Barind sheep of Bangladesh. J Earth Environ Sci. 2020;4(192):2577–0640.

[28] Ma’aruf B, Ibrahim T, Umar H, Shu’aibu A, Okoh J. Effects of age and sex on body weight, body condition score and biometric traits of Yankasa sheep. Niger J Anim Sci Technol. 2018;1:39–46.

[29] Arsalan M, Ullah MA, Waheed A. Application of CHAID algorithm for the identification of morphological traits of indigenous sheep body weight: Significant traits of body weight by CHAID analysis. Proceed Pakistan Acad Sci: A Physical and Comput Sci. 2020;57(2):75–80.

[30] Hoda A, Mecaj R, Hykaj G. Morphometric characterization of four sheep breeds reared in South East of Albania. Poljoprivreda i Sumarstvo. 2022;68(3):241–53.

[31] Alemayehu A, Mirkena T, Melesse A, Getachew T, Haile A. Genetic parameters and trends of growth traits in community-based breeding programs of Abera sheep in Ethiopia. Acta Agriculturae Scandinavica, Section A — Animal Science. 2022;71(1–4):1–11. doi: 10.1080/09064702.2022.2067590

[32] Mohammadi Y, Mokhtari MS, Saghi DA, Shahdadi AR. Modeling the growth curve in Kordi sheep: the comparison of non-linear models and estimation of genetic parameters for the growth curve traits. Small Ruminant Research. 2019;177:117–23. doi: 10.1016/j.smallrumres.2019.06.012

[33] Mathapo MC, Mugwabana TJ, Tyasi TL. Prediction of body weight from morphological traits of South African non-descript indigenous goats of Lepelle-Nkumbi Local Municipality using different data mining algorithm. Trop Anim Health Prod. 2022;54(2):102. doi: 10.1007/s11250-022-03096-9 35152324

[34] Ali A, Javed K, Zahoor I, Anjum KM. Analysis of non-genetic and genetic influences underlying the growth of Kajli lambs. SA J An Sci. 2020;50(4):613–25. doi: 10.4314/sajas.v50i4.13

[35] Yasmin I, Iqbal R, Liaqat A, Khan WA, Nadeem M, Iqbal A, et al. Characterization and comparative evaluation of milk protein variants from Pakistani dairy breeds. Food Sci Anim Resour. 2020;40(5):689–98. doi: 10.5851/kosfa.2020.e44 32968722 PMC7492176

[36] Iqbal F, Waheed A, Huma Z-, Faraz A. Nonlinear growth functions for body weight of thalli sheep using bayesian inference. Pak J Zoo. 2019;51(4). doi: 10.17582/journal.pjz/2019.51.4.1421.1428

[37] Ghiasi H, Lupi TM, Mokhtari MS. The estimation of genetic parameters for growth curve traits in Raeini Cashmere goat described by Gompertz model. Small Ruminant Research. 2018;165:66–70. doi: 10.1016/j.smallrumres.2018.06.015

[38] Kridli RT, Abdullah AY, Obeidat B, Qudsieh R, Titi H, Awawdeh M. Seasonal variation in sexual performance of Awassi rams. Animal Rep. 2018;4(1):38–41.

[39] Anila H, Hajno L. Body measurements of Bardhoka sheep breed from Albania. Acta Biologica Turcica. 2021;34(3):122–7.

[40] Perez ZO, Ybañez AP, Ybañez RHD, Sandoval JFGJ. Body weight estimation using body measurements in goats (Capra hircus) under field condition. Philippine J Vet Animal Sci. 2016;42(1).

[41] Kumar S, Dahiya SP, Dahiya ZS, Patil CS. Prediction of body weight from linear body measurements in sheep. IJAR. 2017;(of). doi: 10.18805/ijar.b-3360

[42] Adjibode G, Tougan U, Daouda I, Mensah G, Youssao A, Hanzen C. Factors affecting reproduction and growth performances in West African Dwarf sheep in sub-Saharan Africa. Int J Agron Agricul Res. 2017;11(1).

[43] Tohidi R, Ismailjami Y, Javanmard A. Analysis of the environmental factors affecting the growth traits of iran-black sheep. Int J Environ Agricul Biotech. 2016;2(1):159–64. doi: 10.22161/ijeab/2.1.21

[44] Kumar DAP, Prakash MG, Gupta BR, Raghunandan T, Chandra AS. Post-weaning growth performance in Deccani sheep. The Pharma Innov. 2017;6(9, Part A):9.

[45] Ganesan R, Dhanavanthan P, Balasubramanyam D, Kumarasamy P. Growth modeling and factors affecting growth traits in Madras red Sheep. Ind J Anim Rese. 2015;49(1):20. doi: 10.5958/0976-0555.2015.00004.7

[46] Niaz T, Kaleri HA, Kaleri RR, Kaleri A, Kaleri AH, Shah RA. Effect of genetic and environment factors on some productive traits of bibrik sheep. Sci Int. 2017;29:577.

[47] Abdul Sattar S, Hubdar Ali K, Gul M, Rameez Raja K, Asma K, Muhammad Akram S, et al. Effect of genetic parameters on some growth performance traits of harnai sheep. J Basic Appl Sci. 2017;13:60–2. doi: 10.6000/1927-5129.2017.13.11

[48] Tec Canche JE, Magaña Monforte JG, Segura Correa JC. Environmental effects on productive and reproductive performance of Pelibuey ewes in Southeastern México. J Appl Animal Res. 2016;44(1):508–12.

[49] Singh G, Kumar S. Circadian activity in different photoperiod (12L: 12 D) and (8L: 16D) in passerine finches. Avian Biology Research. 2022;15(2):68–72. doi: 10.1177/17581559221075982

[50] Yakubu A. Application of regression tree methodology in predicting the body weight of Uda sheep. Scientific Papers Animal Science and Biotech. 2012;45(2):484.

[51] Chitra R, Rajendran S, Prasanna D, Kirubakaran A. Prediction of body weight using appropriate regression model in adult female Malabari goat. Vet World. 2012;5(7):409. doi: 10.5455/vetworld.2012.409-411

[52] Hojjati F, Ghavi Hossein-Zadeh N. Comparison of non-linear growth models to describe the growth curve of Mehraban sheep. J Applied Animal Res. 2017;46(1):499–504. doi: 10.1080/09712119.2017.1348949

